# Correlations Between Intestinal Microbial Community and Hematological Profile in Native Tibetans and Han Immigrants

**DOI:** 10.3389/fmicb.2021.615416

**Published:** 2021-06-21

**Authors:** Yan Ma, Qin Ga, Ri-Li Ge, Shuang Ma

**Affiliations:** ^1^Research Center for High Altitude Medicine, Qinghai University Medical College, Xining, China; ^2^Key Laboratory for Application of High Altitude Medicine in Qinghai Province, Qinghai University, Xining, China; ^3^Qinghai-Utah Joint Research Key Lab for High Altitude Medicine, Qinghai University Medical College, Xining, China

**Keywords:** fecal microbiota, high-altitude adaptation, Tibetan, Han immigrant, hematological profile

## Abstract

Hematological features are one of the best-known aspects of high-altitude adaptation in Tibetans. However, it is still unclear whether the intestinal microbiota is associated with the hematology profile. In this study, routine blood tests and 16S rRNA gene sequencing were used to investigate the differences in the intestinal microbiota and hematological parameters of native Tibetan herders and Han immigrants sampled at 3,900 m. The blood test results suggested that the platelet counts (PLTs) were significantly higher in native Tibetans than the Han immigrants. The feces of the native Tibetans had significantly greater microbial diversity (more different species: Simpson’s and Shannon’s indices) than that of the Han immigrants. The native Tibetans also had a different fecal microbial community structure than the Han immigrants. A Bray–Curtis distance-based redundancy analysis and envfit function test showed that body mass index (BMI) and PLT were significant explanatory variables that correlated with the fecal microbial community structure in native Tibetans. Spearman’s correlation analysis showed that *Megamonas* correlated positively with BMI, whereas *Bifidobacterium* correlated negatively with BMI. *Alistipes* and *Parabacteroides* correlated positively with the PLT. *Succinivibrio* correlated positively with SpO_2_. *Intestinibacter* correlated negatively with the red blood cell count, hemoglobin, and hematocrit (HCT). *Romboutsia* correlated negatively with HCT, whereas *Phascolarctobacterium* correlated positively with HCT. A functional analysis showed that the functional capacity of the gut microbial community in the native Tibetans was significantly related to carbohydrate metabolism. These findings suggest that the hematological profile is associated with the fecal microbial community, which may influence the high-altitude adaptation/acclimatization of Tibetans.

## Introduction

The Tibetan Plateau is both the largest and highest plateau in the world, with a mean elevation of 4,500 m (Zhao et al., 2019), and is therefore considered as the “roof of the world.” It has special environmental conditions, such as low atmospheric pressure, low temperature, low relative humidity, and high solar radiation (Zhao et al., 2018). On the Tibetan Plateau, the main indigenous population is the Tibetans, whereas the Han population constitutes the majority of the immigrant population. Han individuals and other unacclimatized or susceptible people who ascend to altitudes >2,500 m can experience physiological responses during rapid ascent that may be life-threatening (Roach et al., 2018). Increased red blood cell (RBC) counts, packed cell volumes, or high hemoglobin concentrations are frequently observed in physiological responses to high altitude. Patients with chronic mountain sickness are characterized by excessive erythrocytosis (Moore et al., 2010). Hypoxia also alters the proteome of platelets, favoring a prothrombotic phenotype, increasing platelet reactivity, which increases the risk of thrombotic diseases in chronically hypoxic individuals (Rocke et al., 2018). However, Tibetans have been living at high altitudes for thousands of years (Meyer et al., 2017), have established perfectly adapted mechanisms for high-altitude living, and display a suite of adaptive physiological traits: increased resting ventilation, reduced arterial oxygen content, blunted hypoxic ventilation response and pulmonary vasoconstriction response, and a reduced incidence of low birth weight (Jianguo et al., 2002; Beall, 2006, 2007; Tianyi and Kayser, 2006). Lowland individuals (Han) can acclimatize to altitude to mitigate the effects of high-altitude exposure, and during this acclimatization, most changes occur over a period of days up to a few weeks (Muza et al., 2010; West et al., 2012; Qian-Qian et al., 2013). This process does not restore the individual’s performance to that at sea level, nor to one that is completely the same as that of the native population (Yang et al., 2018). Although many genome-wide association studies have identified differences in several genes, such as endothelial PAS domain containing protein 1 (*EPAS1*) and egl-9 family hypoxia inducible factor (*EGLN1*), which are responsible for the genetic adaptation of native highlanders (Simonson et al., 2010; Lorenzo et al., 2014; Arciero et al., 2018; Storz, 2021), limited information is available on the association between the intestinal microbiome, or “second genome” (Grice and Segre, 2012), and the hematology of these natives. Trillions of microorganisms inhabit the human body, strongly colonizing the gastrointestinal tract and outnumbering our own cells (Sohail et al., 2019). Most microbes reside in the gastrointestinal tract, have coevolved with their hosts, and have profound effects on human physiology and nutrition (Turnbaugh et al., 2006; Tremaroli and Bäckhed, 2012).

Short or chronic exposure of humans or animals to hypoxia (the most typical characteristic of high altitude) can affect the composition of the intestinal microbiota (Brigitta et al., 2005; Atanu et al., 2013; Lucking et al., 2018; Zhang et al., 2019). It appears that some indigenous high-altitude humans and animals tend to carry several common genes and some common intestinal microbes that may be related to their adaptation to altitude (Ge et al., 2013; Kang et al., 2016; Li et al., 2016, 2018; Zhang et al., 2016a,b; Lan et al., 2017; Arciero et al., 2018; Bai et al., 2018). These findings suggest that the host genome and the intestinal microbiome have coevolved under the selection pressure exerted by high altitude.

Among the majority populations on the Tibetan Plateau, the native Tibetan and Han immigrants living at same high altitude display significant differences in compositions of their intestinal microbiota (Kang et al., 2016). This is also true of members of the same ethnic group living at different altitudes (Lan et al., 2017). Diet, body mass index (BMI), lifestyle, and age also influence the community composition and structure of the intestinal microbial in native Tibetans and Han immigrants (Li and Zhao, 2015; Lan et al., 2017; Li et al., 2018).

Recent studies have indicated that the intestinal microbiota not only regulates mucosal immunity but also contributes to hematopoiesis. The gut microbiota sustains hematopoiesis. Hematopoietic changes are associated with a significant contraction of the fecal microbiome and are partially rescued by the transfer of fecal microbiota. Platelet counts (PLTs) are consistently and significantly increased in antibiotic-treated mice compared with those in control mice (Josefsdottir et al., 2017; Staffas et al., 2018; Han et al., 2021). Metabolites from the intestinal microbiota, such as short-chain fatty acids (SCFAs), contribute to the production of hematopoietic precursors in specific-pathogen-free (SPF) mice (Trompette et al., 2014). Some of the best-known aspects of high-altitude acclimatization and adaptation involve the hematological system, such as the changes in RBC numbers per unit volume, the hemoglobin (Hb) concentration, and the PLT or activation (West et al., 2012; Shang et al., 2019). However, the association between the intestinal microbiota and the hematological parameters of natives and immigrants living at high altitudes is still unclear. This prompted us to investigate the composition and diversity of the intestinal microbiota in native Tibetans and Han immigrants, and the potential relationship between their intestinal microbial profiles and host hematological parameters during exposure to high altitude.

## Materials and Methods

### Ethics Statement

All the experiments were approved by and performed in accordance with the guidelines and regulations of the Ethics Committee of Qinghai University, Xining, China. Written informed consent was obtained from all the participants and submitted to the Ethics Committee.

### Subjects

The native Tibetan herders (*n* = 26) were living at an altitude of 3,900 m in Da Ri county (33°45'3.83 N, 99°39' E), Guoluo Tibetan Autonomous Prefecture, Qinghai–Tibet Plateau. The Chinese Han immigrants (*n* = 5) had migrated to Da Ri earlier than a year ago. All the enrolled subjects were healthy, with no history of gastrointestinal disease, liver disease, hypertension, or diabetes, as demonstrated by their medical histories and physical examinations.

### Blood Sample Collection and Analysis

Venous blood samples (2 ml) were collected with venipuncture into an EDTA-K2 anticoagulant Vacutainer tubes and then analyzed with BM-2300 fully automated blood cell analyzer (Mindray, Shenzhen, China) within 30 min of the blood drawing.

### Fecal Sample Collection

Approximately 5 g of fresh feces was collected from each participant and placed in two sterile 5-ml tubes, which were immediately transferred to liquid nitrogen and then stored at −80°C.

### Fecal Microbiome Analysis

#### DNA Extraction

The total genomic DNA from each fecal sample was extracted with the PowerFecal™ DNA Isolation Kit (Mo Bio Laboratories, Carlsbad, CA, United States), according to the manufacturer’s instructions. The DNA purity was assessed on 1% agarose gels. The DNA purity and concentration were also calculated with an optical density (OD) analysis at wavelengths of 260 and 280 nm, as the OD_260_/OD_280_ ratio, with a NanoPhotometer^®^ spectrophotometer (Implen, Munich, Germany). The DNA concentrations were measured with the Qubit^®^ dsDNA Assay Kit in a Qubit^®^ 2.0 Fluorometer (Life Technologies, Camarillo, CA, United States).

#### 16S rRNA Gene Sequencing and Data Analysis

16S rRNA gene sequencing was performed by Novogene Bioinformatics Technology Co., Ltd., China. Briefly, the DNA samples were diluted to 1 ng/μl in sterile water and then PCR amplified with the 515F/806R primer set (515F: 5'-GTGCCAGCMGCCGCGGTAA-3', 806R: 5'-XXXXXXGGACTACHVGGGTATCTAAT-3'), which targets the V4 region of the bacterial 16S rRNA. The reverse primer contained a 6-bp error-correcting barcode unique to each sample. All PCRs were performed with Phusion^®^ High-Fidelity PCR Master Mix (New England Biolabs [Beijing] Ltd., Beijing, China). The PCR products were mixed with the same volume of 1 × loading buffer (containing SYB Green dye) and separated electrophoretically on 2% agarose gels for confirmation. Samples with bright major bands of 400–450 bp were isolated for further analysis. All the PCR products were purified with the GeneJET Gel Extraction Kit (Thermo Scientific, Waltham MA, United States). Sequencing libraries were generated with the TruSeq^®^ DNA PCR-Free Sample Preparation Kit (Illumina, San Diego, CA, United States), according to the protocol described by the manufacturer, and index codes were added. After the quality of the libraries was assessed, they were sequenced on the Illumina HiSeq 2500 platform (Illumina), and 250-bp paired-end reads were generated.

### Data Analysis

Student’s *t*-test was used to test the significance of differences in age, gender, height, weight, BMI, oxyhemoglobin saturation measured with a pulse oximeter (SpO_2_), RBCs, Hb, hematocrit (HCT), and PLT between the native Tibetans and Han immigrants.

After 16S rRNA gene sequencing, the paired-end reads were merged with FLASH (Magoč and Salzberg, 2011). The raw tags were quality filtered under specific filtering conditions to obtain high-quality clean tags (Bokulich et al., 2013), according to the QIIME quality-controlled process (Caporaso et al., 2010). The tags were then compared with the reference database (Genomes OnLine Database, GOLD)^1^ using the UCHIME algorithm (Edgar et al., 2011), to detect chimeric sequences, which were then removed (Haas et al., 2011). Uparse (Edgar, 2013) was used to identify the operational taxonomic units (OTUs) by constructing an OTU table. Sequences with ≥97% similarity were assigned to the same OTU. Representative sequences for each OTU were screened for further annotation. The Green Gene Database (Desantis et al., 2006) was used with the RDP Classifier algorithm (Wang et al., 2007) to annotate each representative sequence with taxonomic information. Multiple sequence alignments were constructed with MUSCLE (Edgar, 2004). The numbers of common and unique OTUs were presented in Venn diagrams using BioVenn.^2^ Student’s *t*-test was used to test the significance of differences in the relative abundances of Firmicutes and Bacteroidetes. The Mann–Whitney *U* test was used to test the significance of differences in unclassified *Prevotellaceae*, *Bacteroides*, *Faecalibacterium*, and the Firmicutes/Bacteroidetes (F/B) ratio between the groups. The Mann–Whitney *U* test was used to test the significance of differences in Ace, Chao 1, Simpson’s index, and Shannon’s index between groups. The differences in the overall community compositions and structures of both groups were visualized with the nonmetric multidimensional scaling (NMDS) ordination plots of Jaccard and with Bray–Curtis distance matrices. A MetaStats analysis was used to identify bacterial taxa differentially represented in the groups at the genus or higher taxonomic levels, and values of *p* were corrected with the Benjamini–Hochberg false discovery rate method to determine the values of *q* (White et al., 2009). The linear discriminant analysis (LDA) effect size (LEfSe), which takes into account both statistical significance and biological relevance, was used to search for taxa whose relative abundances differed significantly between the groups, with a default LDA of four (Segata et al., 2011). A Bray–Curtis distance-based redundancy analysis (dbRDA; Clarke and Ainsworth, 1993) and the variance inflation factor (Sheik et al., 2012) function in the “vegan” package of R were used to evaluate the linkages between the fecal microbiota and environmental attributes. The influences of environmental factors on the distributions of genera were calculated with the “envfit” function, which uses multivariate ANOVA for categorical variables and linear correlations for continuous variables. Correlations between age, BMI, SpO_2_, RBC, Hb, HCT, PLT, and the bacterial communities were assessed with Spearman’s correlation analysis (Segata et al., 2011) using the “pheatmap” package in R.

The functional profiles of the microbial communities were predicted with Tax4Fun (AβHauer et al., 2015) with the nearest-neighbor method, based on the minimum 16S rRNA sequence similarity. The Kyoto Encyclopedia of Genes and Genomes (KEGG) database prokaryotic whole-genome 16S rRNA gene sequence was extracted and aligned with the SILVA SSU Ref NR database using the BLASTN algorithm to establish a correlation matrix. The prokaryotic whole-genome functional information from the KEGG database, annotated by UProC and PAUDA, was mapped to do the SILVA database function annotation. The sequenced samples were clustered out of the OTU using the SILVA database sequence as the reference sequence to obtain functional annotation information.

## Results

### Sample Characteristics

In this study, we compared the age, sex, height, weight, BMI, and hematological parameters of native Tibetans and the Han immigrants. The native Tibetans had significantly higher PLT than the Han immigrants (*p* < 0.05; Table 1), whereas age, sex, height, weight, BMI, SpO_2_, RBC, Hb, and HCT did not differ significantly between the two groups (*p* > 0.05; Table 1). The main food of the native Tibetans was roasted barley flour, buttered tea, cheese, and meat, whereas the daily staples of the Han immigrants were noodles, rice, and vegetables.

**Table 1:** Characteristic of the native Tibetans and Han immigrants in Da Ri.

	Native Tibetan	Immigrant Han	Value of *p*
N	26	5	
Age (years)	42.73 ± 18.09	48.2 ± 1.30	0.511
Sex (male/female)	19/7	4/1	1.000
Height (cm)	162.96 ± 8.67	165.40 ± 7.23	0.561
Weight (kg)	62.57 ± 11.85	58.80 ± 9.60	0.509
BMI	19.12 ± 2.95	17.78 ± 2.89	0.359
SpO_2_ (%)	83.54 ± 6.56	86.00 ± 1.87	0.418
RBC (10*12/L)	5.65 ± 0.86	5.17 ± 0.81	0.263
Hb (g/L)	162.46 ± 26.61	156.40 ± 22.76	0.638
HCT (%)	58.60 ± 12.45	56.86 ± 8.29	0.768
PLT (10*9/L)	257.34 ± 61.02	189.40 ± 57.22	0.029

*Values are means ± SD*. *Data were analyzed with the Student’s t-test to compare the variables between the groups. BMI, body mass index; SpO_2_, oxyhemoglobin saturation measured with a pulse oximeter; RBC, red blood cells; Hb, hemoglobin; HCT, hematocrit; and PLT, platelet count*.

### HiSeq Sequencing

The fecal microbiotal compositions were determined by sequencing the 16S rRNA gene in 31 samples from the two groups with the HiSeq platform. A total of 2,674,262 raw reads were obtained. After the low-quality sequences, chimeric sequences, and those that were not classified as bacteria were removed, 2,392,645 sequences remained, with an average number of 77,182 sequences per sample (range 46,224–2,392,645) and an average read length of 253 bp. A clustering analysis assigned the microbial sequences from the samples that shared 97% similarity to the same OTUs. In total, 2,205 OTUs were identified. Rarefaction curves showed that a plateau level was reached in all samples (Supplementary Figure S1), indicating that our sequencing depth was sufficient, and that those additional sequences may identify some rare bacterial species.

### Taxonomic Analysis

**Figure 1:**
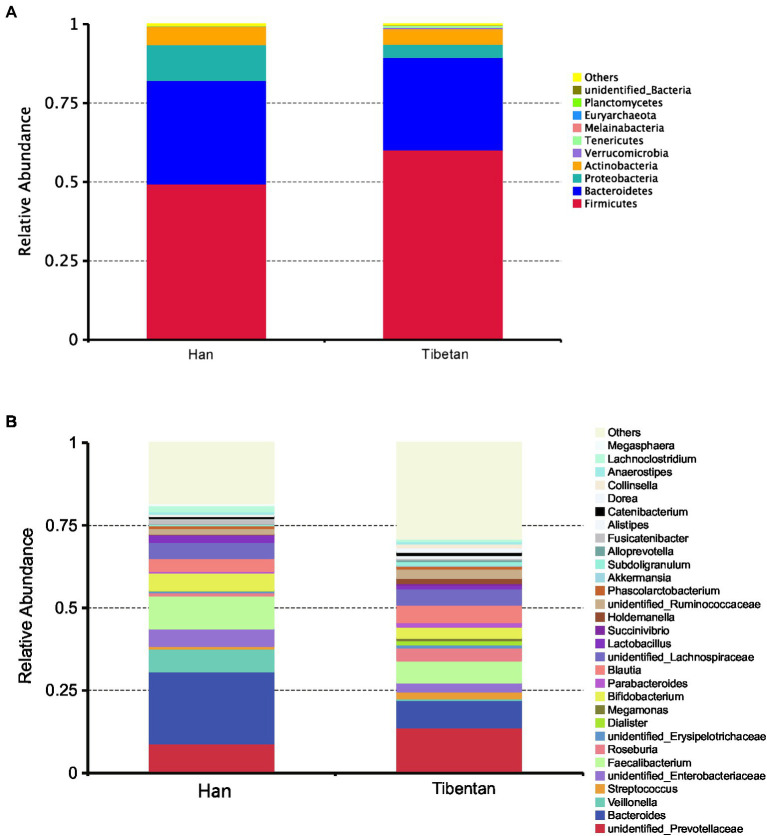
Community composition of fecal microbiota in native Tibetans and Han immigrants. In the stacked bar chart, each bar represents the average relative abundance of each bacterial taxon. The taxa with high relative abundances at the phylum (top 10, **A**) and genus levels (top 30, **B**) are shown.

Of the total OTUs, 970 (43.9%) were common to the native Tibetans and Han immigrants, whereas 961 OTUs (43.5%) were unique to the native Tibetans, and 274 OTUs (12.4%) were unique to the Han immigrants. The fecal microbiota compositions of each group at phylum and genus levels are shown in Figure 1. The fecal microbiotas of the native Tibetans and Han immigrants were dominated by the following phyla: Firmicutes (49.5 and 60.2%, respectively), Bacteroidetes (32.8 and 29.3%, respectively), Proteobacteria (11.3 and 4.3%, respectively), and Actinobacteria (5.7 and 4.9%, respectively). Other rare phyla (mean relative abundance <1%) included Verrucomicrobia, Tenericutes, Melainabacteria, Euryarchaeota, and Planctomycetes. We found no significant differences in the relative abundances of Firmicutes and Bacteroidetes, or in the F/B ratios (Ruth et al., 2005) of the native Tibetans and Han immigrants (*p* > 0.05; Supplementary Table S1). At the genus level, 449 genera were detected. The four core taxa of the fecal bacteria in the native Tibetans were *unclassified Prevotellaceae* (13.69%), *Bacteroides* (8.30%), *Faecalibacterium* (6.62%), and *Blautia* (5.31%). The four core taxa of fecal bacteria in the Han immigrants were *Bacteroides* (21.79%), *Faecalibacterium* (9.96%), *unclassified Prevotellaceae* (8.85%), and *Veillonella* (6.91%). The relative abundance of *unclassified Prevotellaceae* was higher in the native Tibetans than in the Han immigrants, whereas the abundance of *Bacteroides* was higher in the Han immigrants than in the native Tibetans, but the differences were not statistically significant (*p* > 0.05; Supplementary Table S2).

### α-Diversity Analysis

Four α-diversity measures were calculated: Simpson’s index, Shannon’s index, the abundance-based coverage estimator (Ace) index, and Chao 1. We found no significant differences in community richness (Ace and Chao 1) between the native Tibetans and the Han immigrants (*p* > 0.05; Table 1), but the native Tibetan feces had significantly greater microbial diversity (more different species, Simpson’s and Shannon’s indices) than that of the Han immigrants (*p* < 0.05; Table 2).

**Table 2:** Fecal microbial diversity and richness in the native Tibetans and Han immigrants.

Group	Shannon’s index	Simpson’s index	Ace	Chao 1
Native Tibetan	5.53 ± 0.43	0.94 ± 0.01	644.52 ± 151.59	641.37 ± 150.44
Immigrant Han	4.60 ± 0.62	0.88 ± 0.40	645.59 ± 194.35	639.40 ± 190.97
Value of *p*	0.001	0.001	0.856	0.658

*Values are means ± SD*. *Differences between the groups were analyzed with the Mann–Whitney U test*.

### β-Diversity Analysis

**Figure 2:**
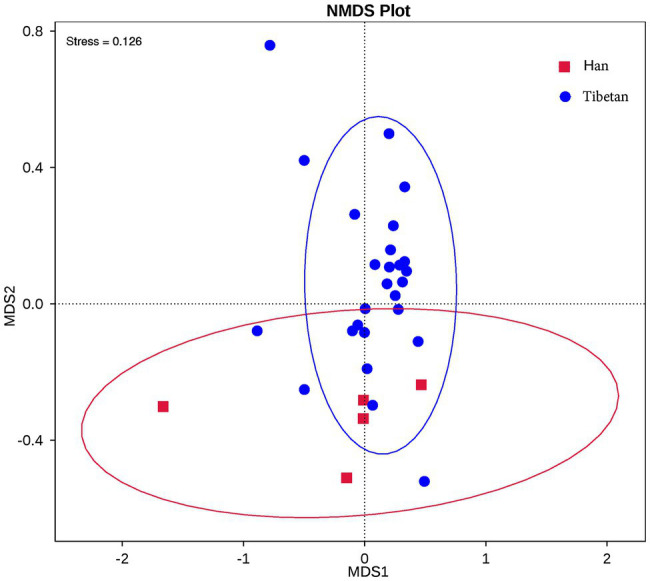
Nonmetric multidimensional scaling (NMDS) showed distinct fecal bacterial communities in the native Tibetans and Han immigrants. Each symbol represents the fecal microbiota of one sample. NMDS plots were derived from the Bray–Curtis distances between the groups.

The relationship between the community structures of the microbiota of the native Tibetans and Han immigrants was examined with NMDS ordination plots, which revealed clear differences in the community compositions and structures of the two groups (Figure 2).

### Differences in Fecal Microbiota of Native Tibetans and Han Immigrants

Of the total OTUs, 961 OTUs (43.5%) were unique to the native Tibetans, and 274 OTUs (12.4%) were unique to the Han immigrants.

**Figure 3:**
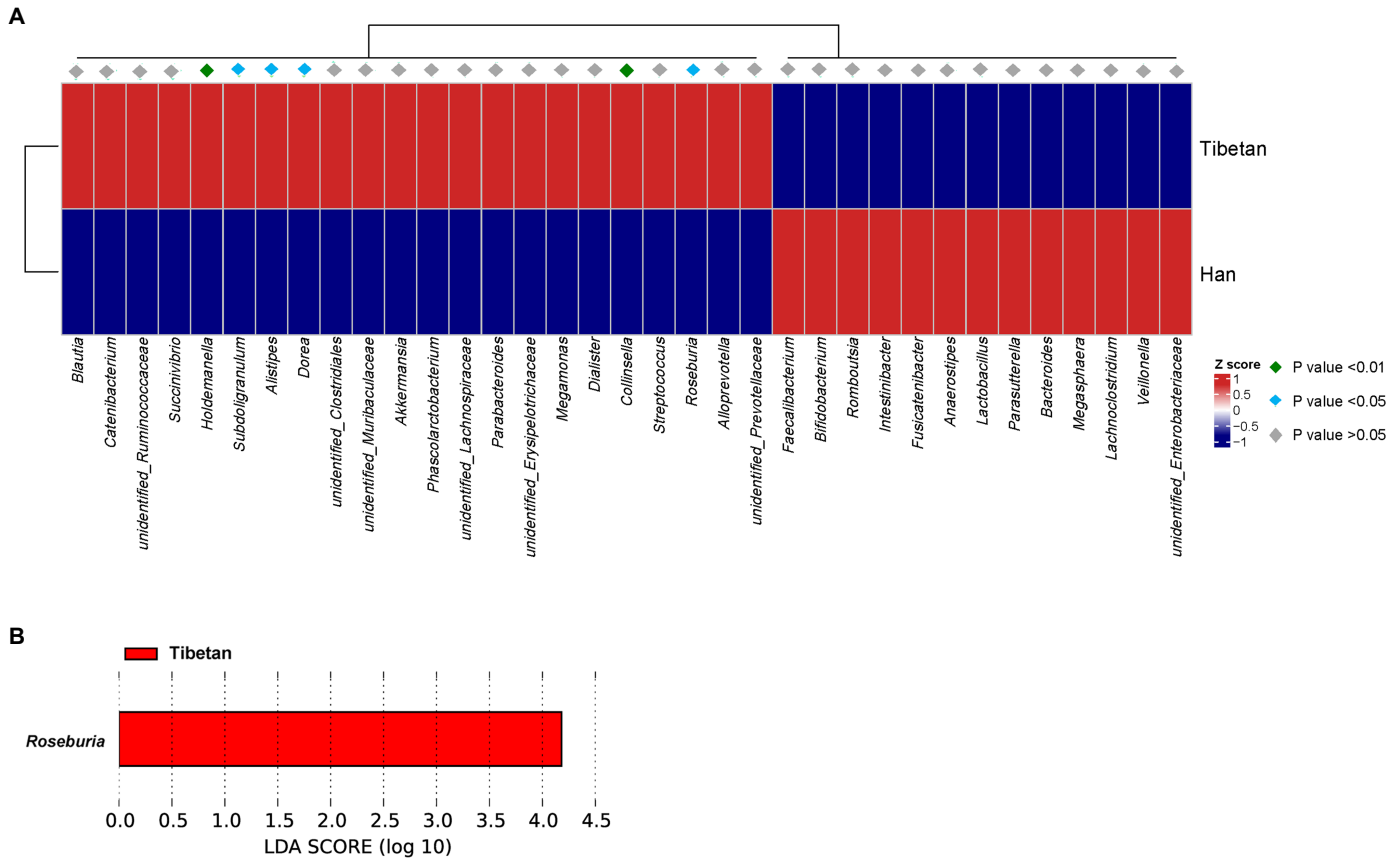
Bacterial taxa that differed significantly between native Tibetans and Han immigrants were identified with: **(A)** a MetaStats analysis, in which the abundances of the 35 most significantly different taxa in the two groups are shown in a heatmap, *p* < 0.05, *q* < 0.05; **(B)** a linear discriminant analysis (LDA) effect size (LEfSe), using the default parameters. A histogram of the LDA scores computed for the bacterial taxa that differed in abundances in the two groups is shown.

A MetaStats analysis of the most-abundant taxa was used to identify the bacterial taxa that differed significantly between the two groups. Figure 3A shows that six bacterial taxa were significantly more abundant (*p* < 0.05) in the fecal microbial of the native Tibetans (*Holdemanella*, *Subdoligranulum*, *Alistipes*, *Dorea*, *Collinsella*, and *Roseburia*) than in that of the Han immigrants. An LEfSe analysis was also conducted to detect the bacterial taxa that differed significantly between the groups. *Roseburia* was significantly more abundant in the fecal microbiota of the native Tibetans than in that of the Han immigrants (Figure 3B).

### Correlations Between Fecal Microbiota and Age, BMI, SpO_2_, RBC, Hb, HCT, and PLT

**Figure 4:**
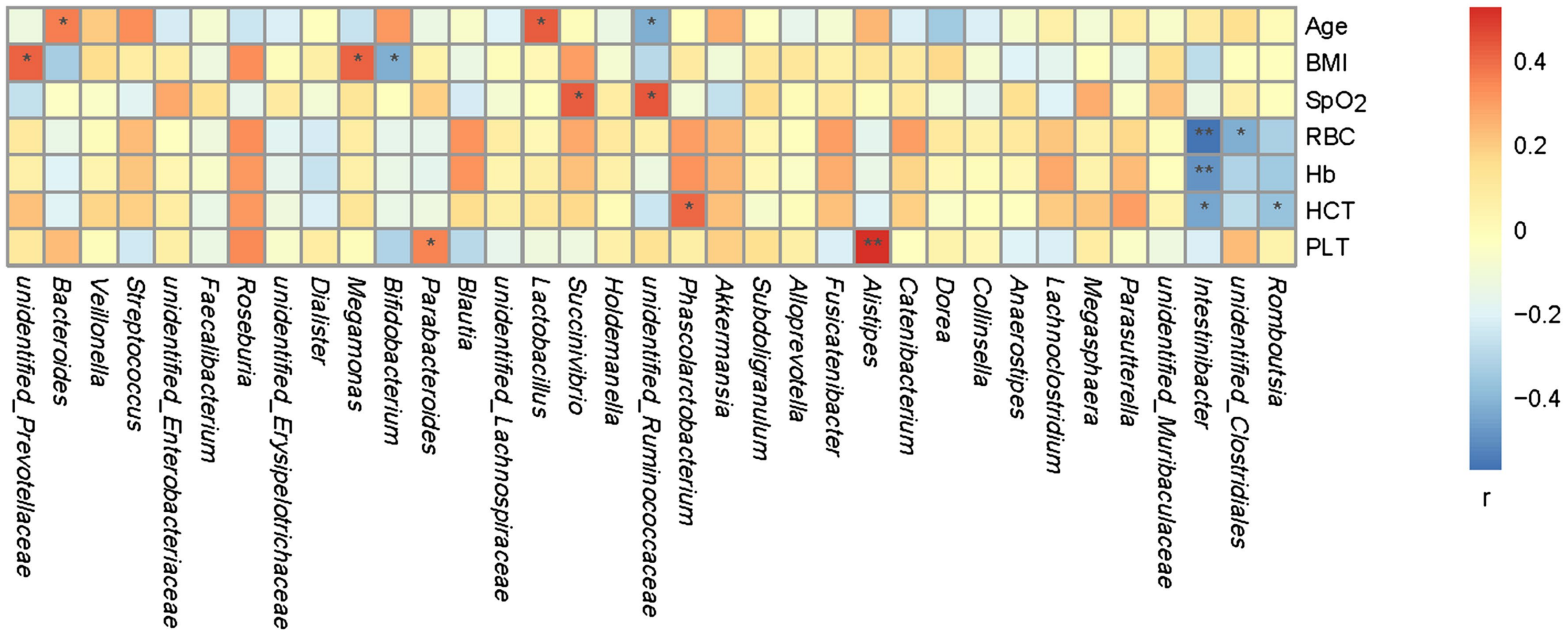
Spearman’s association analysis of bacterial genera and age, BMI, SpO_2_, RBC, Hb, HCT, and PLT. *r* indicates Spearman’s correlation coefficient. Cells are colored based on the value of *r* between significantly altered genera and age, body mass index (BMI), oxyhemoglobin saturation measured with a pulse oximeter (SpO_2_), red blood cells (RBC), hemoglobin (Hb), hematokrit (HCT), and platelet count (PLT). Red represents a significantly positive correlation, blue represents a significant negative correlation, and white represents no significant correlation. ^*^p < 0.05; ^**^p < 0.01.

A Spearman’s correlation matrix was generated to examine the correlations between age, BMI, SpO_2_, RBC, Hb, HCT, PLT, and the bacterial genera. As shown in Figure 4, significant associations were identified between the fecal microbiota and age, BMI, SpO_2_, RBC, Hb, HCT, and PLT. A correlation analysis revealed that genus *unclassified Prevotellaceae* correlated positively with BMI (*r* = 0.42, *p* < 0.05); *Megamonas* correlated positively with BMI (*r* = 0.42, *p* < 0.05); *Bifidobacterium* correlated negatively with BMI (*r* = −0.41, *p* < 0.05); *Alistipes* correlated positively with PLT (*r* = 0.52, *p* < 0.01); *Parabacteroides* correlated positively with PLT (*r* = 0.36, *p* < 0.05); *Bacteroides* correlated positively with age (*r* = 0.36, *p* < 0.05); *Lactobacillus* correlated positively with age (*r* = 0.42, *p* < 0.05); *unclassified Ruminococcaceae* correlated negatively with age (*r* = −0.41, *p* < 0.05) and positively with SpO_2_ (*r* = 0.44, *p* < 0.05); *Succinivibrio* correlated positively with SpO_2_ (*r* = 0.43, *p* < 0.05); *unidentified Clostridiales* correlated negatively with RBC (*r* = −0.42, *p* < 0.05); *Intestinibacter* correlated negatively with RBC (*r* = −0.56, *p* < 0.01), Hb (*r* = −0.48, *p* < 0.01), and HCT (*r* = −0.31, *p* < 0.05); *Romboutsia* correlated negatively with HCT (*r* = −0.36, *p* < 0.05); *Phascolarctobacterium* correlated positively with HCT (*r* = 0.40, *p* < 0.05); and *Roseburia* correlated positively with BMI (*r* = 0.33, *p* = 0.06), RBC (*r* = 0.33, *p* = 0.06), Hb (*r* = 0.31, *p* = 0.08), HCT (*r* = 0.31, *p* = 0.08), and PLT (*r* = 0.34, *p* = 0.05).

**Figure 5:**
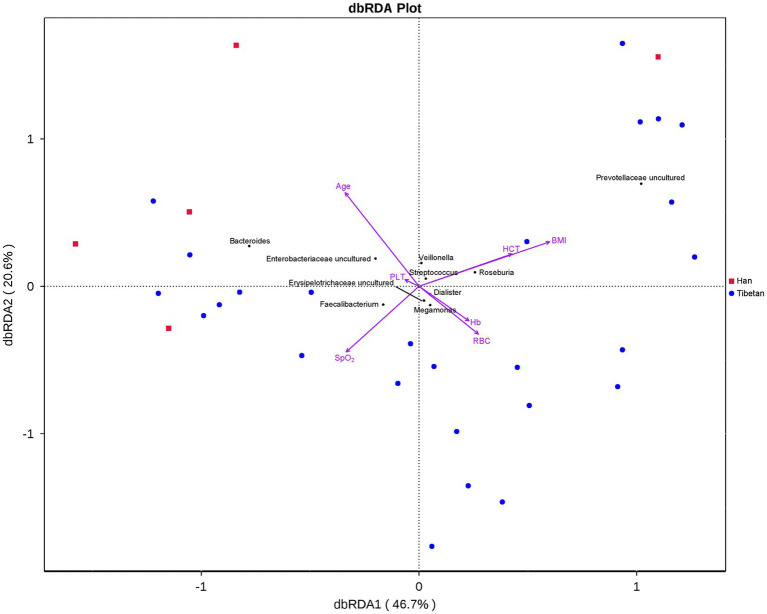
Bray–Curtis distance-based redundancy analysis (dbRDA) of the fecal microbiota and explanatory variables in native Tibetans and Han immigrants.

To determine whether BMI or other variables had an additional effect on the fecal microbial community structure, we performed a Bray–Curtis dbRDA. Age, BMI, SpO_2_, RBC, Hb, HCT, and PLT were associated with the microbial community structure (Figure 5). Furthermore, an envfit function test showed that BMI and PLT were significant explanatory variables (Table 3).

**Table 3:** dbRDA envfit analysis of the correlation between fecal microbiota and explanatory variables in native Tibetans and Han immigrants.

	RDA1	RDA2	*r*^2^	*p*
BMI	0.973172	0.230079	0.308357	0.0059
PLT	−0.04598	0.998943	0.434019	0.0005

*RDA1 and RDA2 are the cosines of the angles between the arrows of the explanatory variables and the sorting axes. r^2^, the goodness of fit statistic, is the squared correlation coefficient*.

### Functional Profiles of Fecal Microbiota in Native Tibetans and Han Immigrants

**Figure 6:**
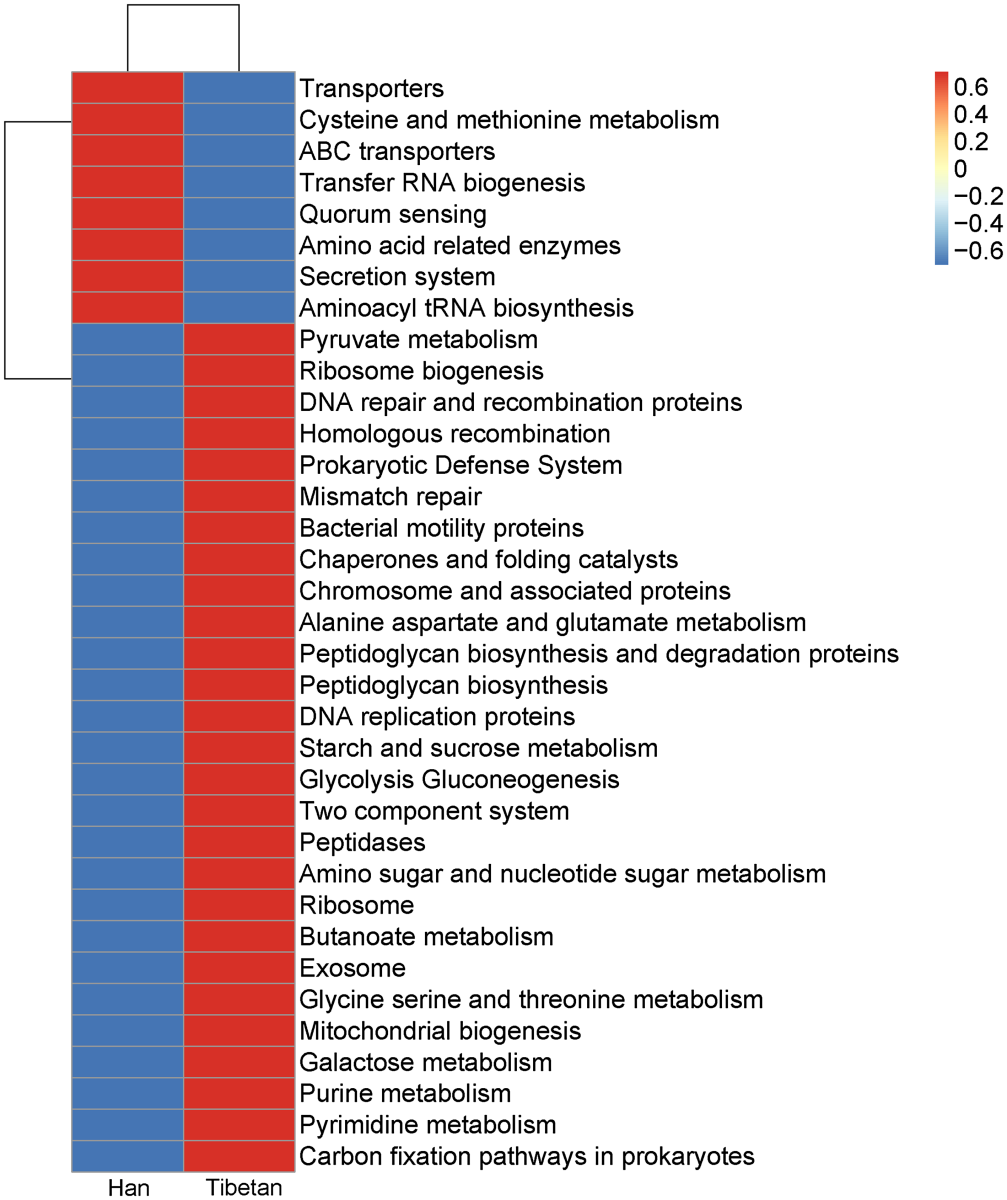
Functional profiles of the fecal microbial communities in native Tibetans and Han immigrants. The 35 most-abundant pathways at the third level of KEGG pathways are shown in a heatmap.

To further investigate the functional capacities of the fecal microbial communities in the native Tibetans and Han immigrants, Tax4Fun was used to examine the functional profiles of the fecal microbiota in the two groups. Figure 6 shows the 35 most-abundant pathways at the third level of KEGG pathways. Of these 35 pathways, 27 were more abundant in the fecal microbial community of the native Tibetans than in that of the Han immigrants. The functional capacities of the intestinal microbial communities in the native Tibetans were enriched in metabolism (pyruvate metabolism; butanoate metabolism; alanine, aspartate, and glutamate metabolism; glycolysis and gluconeogenesis; starch and sucrose metabolism; amino sugar and nucleotide sugar metabolism; glycine, serine, and threonine metabolism; purine and pyrimidine metabolism; and carbon fixation pathways in prokaryotes) and genetic information processing (ribosome biogenesis; DNA repair and recombination proteins; homologous recombination; mismatch repair; chaperones and folding catalysts; chromosome and associated proteins; and DNA replication proteins). Eight pathways (transporters; cysteine and methionine metabolism; ABC transporters; transfer RNA biogenesis; quorum sensing; amino acid related enzymes; secretion system; and aminoacyl tRNA biosynthesis) were enriched in the fecal microbial community of the Han immigrants.

## Discussion

In this study, we focused on the composition, structure, and diversity of the gut microbiota in two ethnic groups (Tibetan and Chinese Han), living at high altitude (Qinghai), and their correlations with some explanatory factors, especially hematological parameters.

Previous studies have shown that altitude and ethnicity affect the intestinal bacterial profiles of Tibetans (Kang et al., 2016; Lan et al., 2017). Diet, BMI, lifestyle, and age also influence the composition and structure of the Tibetans’ intestinal microbial communities (Li and Zhao, 2015; Lan et al., 2017; Li et al., 2018). However, few studies have examined the association between intestinal microbial profiles and hematological parameters. Our results show that age, BMI, SpO_2_, RBC, Hb, HCT, and PLT correlated with the intestinal microbial community structure, and that BMI and PLT were significant explanatory variables. The genus *Megamonas* correlated positively with BMI, whereas *Bifidobacterium* was negatively associated with BMI. The microbiota of obese subjects contain increased numbers of *Megamonas* (Chiu et al., 2014) and reduced numbers of *Bifidobacterium* (Million et al., 2012). In this study, *Alistipes* and *Parabacteroides* correlated positively with PLT. *Parabacteroides species* modulate the host metabolism by the produce of succinate and secondary bile acids (Wang et al., 2019). Bile acid receptors and platelet-activating factor receptors are numbers of the G-protein-coupled receptors family, which are activated by lipid-derived endogenous ligands (Cooper et al., 2017). Cholesterol, cholic acid, ursodeoxycholic acid, deoxycholic acid, and tauroursodeoxycholic acid affect platelet activation through adenosine diphosphate (ADP; Baele et al., 1980; McGregor et al., 1980; Tan et al., 2012). The platelets of highlanders are hyperactive, and the platelet transcriptome and proteome are markedly altered under chronic exposure to high altitudes, together with increased circulating ADP (Shang et al., 2019). *Succinivibrio*, which correlated positively with SpO_2_, ferments carbohydrates and generates succinate, acetate, formate, and lactate (Bryant, 2015). *Intestinibacter* was negatively associated with RBC, Hb, and HCT and is reportedly associated with resistance to oxidative stress (Forslund et al., 2015). *Romboutsia* was negatively associated with HCT, whereas *Phascolarctobacterium* correlated positively with HCT. *Romboutsia* currently includes three species, and *R*. *timonensis* has only been isolated from the right colon of a human with a severe anemia. *Phascolarctobacterium* is a producer of SCFAs, including acetate and propionate. The gut microbiota sustains hematopoiesis (Chen et al., 2020). The harvesting of nutrients from the diet is a well-recognized feature of the symbiotic relationship between the microbiota and its host throughout evolution (Baeckhed et al., 2005). A previous study reported that energy harvested from the diet is a critical mechanism by which the gut microbiota contributes to hematopoiesis after bone-marrow transplantation (Staffas et al., 2018) and hematopoietic stem cell transplantation (Staffas and Brink, 2019).

Altitude may exert an important effect on the human energy balance, and the energy demands of highlanders are high (Kayser and Verges, 2013). High F/B ratios and a high proportion of Firmicutes have been shown to be associated with the highly efficient extraction of energy from food (Turnbaugh et al., 2008). Our results show that there were no differences in the high relative abundances of Firmicutes and Bacteroidetes or in the F/B ratios of the Tibetans and Chinese Han living in the same high-altitude area. Similar results were reported by Li and Zhao (2015), who found that the F/B ratio was higher, Firmicutes was significantly more abundant, and Bacteroidetes was significantly less abundant in the intestinal microbiota of Tibetans and Han immigrants living at high altitudes than in Han subjects living at low altitudes. Interestingly, obese people show similar changes in Firmicutes and Bacteroidetes to those of highlanders with a “normal” BMI according to accepted standards (Tan, 2004; Ley et al., 2006).

In this study, we observed that the genera *Subdoligranulum*, *Roseburia*, *Alistipes*, *Holdemanella*, *Collinsella*, and *Dorea* were significantly more abundant in the fecal bacteria of the native Tibetans than in those of the Han immigrants. *Subdoligranulum* and *Roseburia* produce butyrate from complex carbohydrates and plant polysaccharides (Duncan et al., 2002; Holmstrøm et al., 2004), and butyrate is a major SCFA product from an intestinal source, providing at least 60–70% of the energy requirements of colonocytes (Hamer et al., 2008). SCFAs act not only as energy sources for the colonic epithelium but also as systemic nutrients. SCFA levels can directly affect the substrate and energy metabolism in the peripheral tissues, such as adipose tissues, skeletal muscle, and liver (Canfora et al., 2015). SCFA producers are also responsible for regulating the protection of cells from oxidative stress (Ticinesi et al., 2018). Notably, SCFAs have been shown to contribute to the production of hematopoietic precursors in SPF mice (Trompette et al., 2014). A high-fat diet was associated with an increased abundance of *Alistipes* relative to that associated with a lower-fat diet in healthy young adults in a 6-month randomized, controlled feeding trial (Wan et al., 2019). *Alistipes* is also more abundant in patients with type 2 diabetes (Qin et al., 2012). Tibetans frequently consume high-fat foods, such as buttered tea, cheese, and meat. However, in our study, the Tibetans had normal BMIs and no diabetes. *Holdemanella biformis* is associated with cholesterol, low-density lipoprotein–cholesterol (LDL-C), and free fatty acids (Brahe et al., 2015). The elevated lipid concentration in Tibetans might be attributable to increased lipid synthesis (Ge et al., 2012). *Collinsella* can alter the intestinal absorption of cholesterol, reducing glycogenesis in the liver and increasing triglyceride synthesis, and the abundance of *Collinsella* correlates positively with circulating insulin (Gomez-Arango et al., 2018). Therefore, it is possible that having more *Subdoligranulum*, *Roseburia*, *Alistipes*, *Holdemanella*, and *Collinsella* in the intestine is beneficial for energy capture. The functional capacity of the intestinal microbial communities in the native Tibetans showed that metabolism, especially carbohydrate metabolism (pyruvate metabolism; butanoate metabolism; alanine, aspartate, and glutamate metabolism; glycolysis and gluconeogenesis; starch and sucrose metabolism; and amino sugar and nucleotide sugar metabolism), is one of the most frequent functional classes annotated at the third level of the KEGG pathways. The metabolisms of pyruvate, butanoate, starch, and sucrose are important in the fermenting of nonabsorbed carbohydrates to SCFAs. Carbohydrate metabolism, rather than lipid metabolism, is increased in the Tibetans (Ge et al., 2012). Like Tibetans, Sherpas are also from the Himalayan region and use the oxidation of carbohydrates over the oxidation of intramyocellular lipids and lipid substrates for their energy needs (Holden et al., 1995; Murray, 2009; Gilbertkawai et al., 2014). Interestingly, Sherpa and Aymara, who are also from high altitudes, have different gut microbiota compositions and potential functions. For example, *Treponema*, *Butyrivibrio*, and *RFN20* characterize the gut microbiota of Sherpa and Aymara, and metabolic functions, such as the synthesis and degradation of ketone bodies, vitamin B6 metabolism, the degradation of caprolactam, and the biosynthesis of the terpenoid backbone and unsaturated fatty acids, are elevated in those two populations (Quagliariello et al., 2019). Genetic, geographic, dietary structural, and cultural differences in the hosts may contribute to the diverse gut microbiota of the different high-altitude populations, supplying suitable strategies for each population to cope with its extreme environment.

We also found that the feces of native Tibetans contain significantly greater bacterial diversity than those of the Han immigrants, whereas there was no significant difference in the microbial community richness in the two groups. Increased diversity and redundancy within a microbial community enhance the stability of the ecosystem, including its resistance to acute toxic effects and its general resilience (Konopka, 2009). As the altitude increases, Tibetans display greater intestinal microbial diversity and richness than Tibetans living at lower altitudes (Lan et al., 2017). However, Han individuals living at high altitudes show a reduction in their total intestinal bacteria compared with those of Han individuals living at lower altitudes (Lan et al., 2017). These findings suggest that the more primitive lifestyle of the Tibetans may be associated with the high microbial diversity and richness observed at higher altitudes than is observed at lower altitudes. Urbanization is also related to a loss of intestinal microbial diversity in humans (Winglee et al., 2017), and the urbanization of Tibetan herdsmen has affected the β-diversity of their microbiota community (Li et al., 2018). The functional redundancy of the microbial community could decrease in Han people who migrate to higher altitudes, thus weakening the stability of the ecosystem.

The study subjects were carefully selected, so that the indigenous Tibetans had been living in Da Ri for generations, and the Han individuals had been living in the same region for more than 1 year. And the Han individuals who enrolled were without travel between Da Ri and low altitudes during their residence. It is the major reason that this study had a limited number of samples in the Han immigrants group. Although this preliminary study was performed on a limited sample size, it extends the series of recent studies of the association between the gut microbiota and high-altitude adaptation/acclimatization, and provides valuable information on the fecal microbial communities and hematological profiles in highlanders. Importantly, future studies must be based on larger sample sizes and confirm the interaction between the gut microbiota and hematopoiesis in high-altitude populations.

## Conclusion

Our findings on the compositions and diversity of the fecal microbiota in native Tibetans and Han immigrants living at high altitude indicate that the taxa associated with energy metabolism (such as SCFAs production) were more enriched in the fecal microbiota of the natives Tibetan than that of the Han immigrants. Correspondingly, functional annotations related to carbohydrate metabolism (such as SCFAs-related pathways) were also enriched in the fecal microbiota of the native Tibetans relative to those in the Han immigrants. These bacteria probably allow Tibetans to obtain more energy from food to meet their energy demands at high altitude. BMI, age, SpO_2_, RBC, Hb, HCT, and PLT correlated with the intestinal microbial community structure in the highlanders. Some taxa (such as *Alistipes* or *Parabacteroides*) showed positive or negative associations with BMI and the hematological parameters. Our study provides valuable insights into the possible relationships between the fecal microbial communities and hematological profiles in Tibetans. The mechanisms underlying the effects of the intestinal microbiota on the hematological parameters in highlanders require further large-scale studies. The range of microbial products that signal to the host to influence normal hematopoiesis and the microbial species from which they derive is yet to be clarified.

## Data Availability Statement

The datasets presented in this study can be found in online repositories. The names of the repository/repositories and accession number(s) can be found at FigShare, https://figshare.com/s/f477aa8a35c4ecb9f06a; doi: 10.6084/m9.figshare.14503209.

## Ethics Statement

The studies involving human participants were reviewed and approved by the Ethics Committee of Qinghai University, Xining, China. The patients/participants provided their written informed consent to participate in this study.

## Author Contributions

YM and SM designed the experiments. YM, QG, and SM performed the experiments. YM analyzed the data and wrote the manuscript. R-LG provided financial support. All authors contributed to the article and approved the submitted version.

### Conflict of Interest

The authors declare that the research was conducted in the absence of any commercial or financial relationships that could be construed as a potential conflict of interest.

## Footnotes

^1^http://drive5.com/uchime/uchime_download.html

^2^http://www.biovenn.nl/index.php
