# Supplementary material for: Correlations Between Intestinal Microbial Community and Hematological Profile in Native Tibetans and Han Immigrants
Source: Front Microbiol. 2021 Jun 21;12:615416. doi: 10.3389/fmicb.2021.615416 (PMC8257080; doi:10.3389/fmicb.2021.615416)
Supplement: Supplementary file 1 [file Data_Sheet_1.DOCX]

**Supplemental information**

**Correlations between Intestinal Microbial Community and Hematological Profile in Native Tibetans and Han immigrants**

Yan Ma^1,2,3^, , Qin Ga^1,2,3^, Rili Ge^1,2,3^, Shuang Ma^1,2,3^*

**
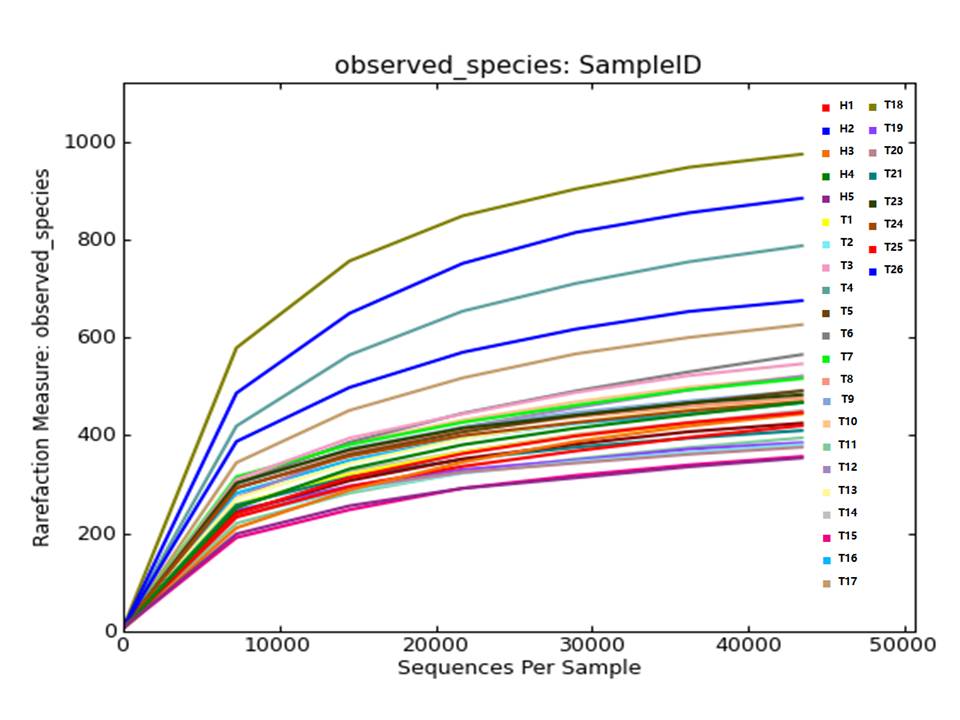
**

**Fig. S1** Rarefaction curves of samples in Tibetans and Han immigrants based on OTUs detected using similarity threshold of 97%.

**Table S1** Proportions of Firmicutes, Bacteroidetes and F/B ratios richness between Tibetans and Han immigrants

| Groups | Relative abundance of Firmicutes | Relative abundance of Bacteroidetes | Firmicutes/Bacteroidetes (F/B) ratios) |
| --- | --- | --- | --- |
| Tibetans | 60.19% ± 13.21% | 29.29% ± 12.99% | 2.14 ±1.78 |
| Han immigrants | 49.45% ± 7.75% | 32.75% ± 15.48% | 2.79 ±1.92 |
| *p* value | 0.092 | 0.600 | 0.480 |

Values are mean±SD.

Student's *t* test was used to test the significance of relative abundance of Firmicutes Bacteroidetes. Mann–Whitney *U* test was used to test the significance of Firmicutes/Bacteroidetes (F/B) ratios between the groups.

**Table S2** Proportions of *Prevotellaceae uncultured*, *Bacteroides*, *Faecalibacterium* richness between Tibetans and immigrant Han

| Groups | Relative abundance of *Prevotellaceae uncultured* | Relative abundance of *Bacteroides* | Relative abundance of *Faecalibacterium* |
| --- | --- | --- | --- |
| Tibetans | 13.69% ± 13.05% | 8.30% ± 9.71% | 6.62% ± 4.10% |
| Han immigrants | 8.85% ± 17.81% | 21.79% ± 17.14% | 9.96% ± 11.19% |
| *p* value | 0.147 | 0.119 | 0.914 |

Values are mean±SD.

Mann–Whitney *U* test was used to test the significance of *Prevotellaceae uncultured*, *Bacteroides*, *Faecalibacterium* between the groups.
